# Eukaryovorous Predation in Evolutionarily Significant Excavate‐Like Flagellates

**DOI:** 10.1111/jeu.70084

**Published:** 2026-04-29

**Authors:** Sei Suzuki‐Tellier, James F. Cass, Elizabeth J. Weston, Kirsty Y. Wan, Alastair G. B. Simpson, Thomas Kiørboe

**Affiliations:** ^1^ Centre for Ocean Life, DTU Aqua, Technical University of Denmark Kongens Lyngby Denmark; ^2^ Living Systems Institute, University of Exeter Exeter UK; ^3^ Department of Biology and Institute for Comparative Genomics Dalhousie University Halifax Nova Scotia Canada

**Keywords:** extrusome, hunter, LECA, motility behavior, prey capture, ventral groove

## Abstract

Accumulating evidence suggests that the Last Eukaryotic Common Ancestor (LECA) resembled contemporary excavates, a non‐monophyletic group of deep branching flagellates. Here we explore the functional ecology of distantly related deep‐branching “excavate‐like” flagellates (in Alveolata and Provora) that share with many excavates a vane‐bearing posterior flagellum beating in association with a ventral groove. This arrangement is key to generating a feeding current in the bacterivorous typical excavates. However, the four species examined here are motile “hunters” that randomly encounter eukaryotic prey. Initial prey attachment may be facilitated by the force generated by the vaned flagellum but eventually by firing extrusomes. Propulsion is driven by the two flagella that are directed backwards. Despite minor differences, the propulsion and hunting strategies are similar between these two deep‐branching groups and distinctly different from those found in typical excavates. While the vane‐and‐groove morphology may help prey attachment, simulations show that it seems energetically disadvantageous to propulsion. Thus, its overall role, origin, and phylogenetic implications remain unclear. This study adds to our knowledge of the functional ecological range of living vane‐ and groove‐bearing flagellates. Given their significance to understanding early eukaryote evolutionary history, this may in turn shed light on the deep history of eukaryote life.

## 1 Introduction

Flagellates, single‐celled organisms equipped with one or a few flagella, are found in all the major branches of the Eukaryotic Tree of Life (Tikhonenkov 2020). Hence, they are key to understanding early eukaryotic evolution. The Last Eukaryotic Common Ancestor (LECA) was a biflagellate, and recent evidence suggests that it had a cell body with a groove shaped by a particular cytoskeletal arrangement, and a flagellum equipped with a vane operating in association with the groove (Williamson et al. 2025). These are morphological traits found in “typical excavate” species from the deep‐branching groups Discoba, Metamonada, and Malawimonadida. Although unified by their morphology (Simpson 2003; Heiss et al. 2013; Suzuki‐Tellier et al. 2023), the paraphyletic relationship between “excavate taxa” has become evident. In particular, several phylogenomic analyses in which eukaryotes are rooted using prokaryotic outgroups place Malawimonadida and Discoba on opposite sides of the root of the tree (He et al. 2014; Derelle et al. 2015; Williamson et al. 2025), implying that the excavate morphology may be ancient and that LECA was an excavate‐like cell.

Despite differences between the “typical excavates” in the three excavate clades, such as the orientation and number of vanes and the beat patterns of the two flagella, their morphology functions in a very similar way: the vaned flagellum generates a feeding current that passes through the groove, and small bacterial prey are captured and phagocytized at the posterior end of the groove (Suzuki‐Tellier et al. 2024). The ventral groove and the vane‐bearing flagellum function jointly in an energetically efficient manner: a vaned flagellum beating in association with a groove requires less power than a naked flagellum to generate the same flow (Suzuki‐Tellier et al. 2024).

A ventral groove in the broad sense is not exclusive to excavates, however. This morphological feature is also present in some form in deep‐branching stramenopiles, ancyromonads, apusomonads (Heiss et al. 2011, 2013; Yubuki and Leander 2013), some members of CRuMs (Brugerolle et al. 2002; Brugerolle 2006), alveolates (Gigeroff et al. 2023; Tikhonenkov et al. 2014), and in the recently described supergroup Provora (Tikhonenkov et al. 2022). Interestingly, some deep‐branching alveolates (among the “colponemids”) and species of Provora also feature a more‐or‐less conspicuous vane (or vanes) on the posterior flagellum, specifically in the portion that parallels the ventral groove. However, in these “excavate‐like” groups, the groove‐and‐vane system seems to function differently than in “typical excavates,” evidenced by the fact that these species are eukaryovores, not bacterivores (Gigeroff et al. 2023; Mylnikov and Tikhonenkov 2009; Tikhonenkov et al. 2014, 2022).

The degree of homology between the flagellar vanes and grooves of provorans, colponemid alveolates and typical excavates is unclear. The well‐characterized underlying microtubular cytoskeleton of the groove of nibbleromonad provorans is mostly similar to that of typical excavates (Belyaev et al. 2024), while lacking the latter's distinctive suite of associated non‐microtubular fibers (Simpson 2003). Meanwhile, the vanes are similar in being supported by internal lamellae (Simpson 2003; Janouškovec et al. 2013; Tikhonenkov et al. 2014, 2022), but the fine structure or other detailed data (e.g., protein composition) has not been compared. Nonetheless, given the inference that vanes and grooves may have co‐evolved early in the evolutionary history of crown eukaryotes, a full understanding of the operation and capabilities of vane‐ and groove‐bearing flagellates would be valuable. At present, the function of the excavate‐like attributes in these unrelated taxa remains poorly documented; resolving this may contribute to clarifying our view of eukaryotic cells from more than a billion years ago.

Here, we study the propulsion and foraging behaviors of *Neocolponema saponarium* (Alveolata), *Colponema vietnamica* (Alveolata), *Nibbleromonas kosolapovi* (Provora), and *Nebulomonas marisrubri* (Provora). We analyze the flagellar behavior, the prey capture and handling processes, and the role of the vaned flagellum together with a groove.

## 2 Materials and Methods

### 2.1 Species and Culturing

The slightly halophilic (growth medium salinity of 50‰) alveolate *Neocolponema saponarium* (Isolate GEM‐Colp) is maintained in the Simpson Lab, Dalhousie University. The freshwater alveolate *Colponema vietnamica* (clone Colp‐tractor) and the two marine provoran species, *Nibbleromonas kosolapovi* (clone Colp‐32) and *Nebulomonas marisrubri* (clone Colp‐4c) were obtained courtesy of Denis V. Tikhonenkov. For clarity, *Neocolponema saponarium*, *Nibbleromonas kosolapovi* and *Nebulomonas marisrubri* are abbreviated respectively as *Neo. saponarium*, *Nib. kosolapovi* and *Neb. marisrubri*, in the text below.

The cultures were maintained by co‐culturing the eukaryovorous flagellates with bacterivorous flagellates that served as prey. An autoclaved cereal grain promoted the growth of bacteria as food for the bacterivorous flagellates. *Neocolponema saponarium* was maintained in a 25 cm^2^ culture flask with 10 mL of the carbonated‐rich medium “CR50” (Mesbah et al. 2007; Gigeroff et al. 2023) (salinity 50‰) and a barley grain, feeding on the recently described kinetoplastid *Novijibodo darinka* (GEM‐Kin; Packer et al. 2025). *Colponema vietnamica* grew in freshwater Pratt medium (Tikhonenkov et al. 2014) with a quinoa grain, feeding on the kinetoplastid *Parabodo caudatus* (BAS‐1, IBIW RAS) in a small Petri dish (Ø 60 mm). The two marine provoran species, *Nib. kosolapovi* and *Neb. marisrubri*, both grew in small Petri dishes with 5 mL of Artificial Sea Water (ASW) with salinity 25‰ and a quinoa grain, feeding on the kinetoplastid *Procryptobia sorokini*. All cultures were kept in the dark at 18°C–20°C. Additionally, *Nib. kosolapovi* and *Neb. marisrubri* were fed the cryptophyte microalga *Rhodomonas* sp., and co‐cultures were kept in a 12:12 light regime at 20°C–22°C.

### 2.2 Transmission Electron Microscopy

To confirm the presence of extrusomes in *Neo. saponarium*, cells were cultured in carbonate rich “CR20” media (salinity 20‰) for 4 days, harvested by centrifugation, and fixed for Transmission Electron Microscopy (TEM) via high‐pressure‐freezing and freeze‐ substitution. A fixation protocol from Yurchenko et al. (2014) was modified by using dilute sterile sea water (salinity 20‰) for rinse steps, using 20% dextran (w/v) as a cryoprotectant, and infiltrating the sample in low viscosity Spurr's resin. Ultra‐thin sections (~60 nm) were cut with a Leica EM UC7 ultramicrotome (Leica Microsystems GmbH, Germany), mounted on pioloform‐coated slot grids, and stained using UranyLess (cat no. 22409, Electron Microscopy Sciences) and Lead Citrate (cat no. 22410, Electron Microscopy Sciences) for 1 min each. Sections were examined on a FEI Tecnai 12 transmission electron microscope and images captured with an AMT Nanosprint15L‐MkII camera (Woburn, Mass. USA). Images were processed post‐capture using Affinity Photo and Affinity Designer (both V2.6.5) (Serif (Europe) Ltd. 1987).

### 2.3 Prey Capture and Swimming Behavior

Individual behaviors (swimming; prey encounter, capture, and handling; flagellar kinematics and wave patterns) were examined in samples prepared with mixtures of predator and prey. Prey sizes varied from somewhat smaller, close to, or larger than the predators (Table S1). The observation chambers consisted of a plastic ring (16 mm inner diameter and 3 mm height) fixed between two glass coverslips that were sealed with Vaseline on the bottom and by surface tension on the top. Chambers were filled with 600 μL of culture and predation behavior was observed with an Olympus IX71 inverted microscope, using an Olympus U Plan Fl N 100X/1.30 oil Ph3 phase‐contrast objective, Olympus LC Plan Fl 40X/0.6 Ph2 phase contrast‐objective, an Olympus LC Plan FL 40X/0.6 objective, or an Olympus U Plan Fl 20X/0.5 Ph1 phase‐contrast objective. Videos were recorded using a high‐speed Phantom Camera (Miro LAB 320, USA) at 25–500 Hz and fields of view ranging between 50 × 50 μm^2^ and 461 × 288 μm^2^ with a resolution of 512 × 512 or 1920 × 1200 pixels. Light intensities were moderate to minimize heating and irradiation effects.

### 2.4 Food‐Dependent Motility Patterns

To examine larger scale‐motility patterns in the absence and presence of food we videorecorded populations of cells at low magnification (20X objective phase contrast; field of view 950 × 600 μm^2^) at 12.5 or 25 Hz. *Neo. saponarium* and *C. vietnamica* were inoculated with their respective well‐established prey cultures, where the grains had been removed 1 day before to limit prey growth, while *Nib. kosolapovi* and *Neb. marisrubri* were fed *Rhodomonas* sp. The predators were recorded after (1) the populations had grown but the concentration of prey was still high and (2) when the prey had been depleted or nearly depleted. Behaviors were recorded in two independent samples on each occasion.

Motility patterns were observed at two focal planes: (1) when swimming or skidding along the surface, where most of the prey was found, and (2) when swimming freely in the fluid without surface interaction. A total of 6 videos per combination of food treatment and focal plane were analyzed.

Swimming tracks were extracted from 75 s long videos using the ImageJ plugin TrackMate (Schneider et al. 2012). For videos focused on the surface, only tracks > 90 frames (7.2 s) long were included; for videos focused in the water column, all tracks were included. Tracks from 6 videos per treatment were combined, and the Root Mean Squared (RMS) distance traveled as a function of time was computed for each treatment.

We hypothesize that starved individuals move in a more directionally persistent (ballistic) pattern than well‐fed individuals. A ballistic pattern is the best strategy to find new, distant food sources, although it also implies a higher risk of themselves encountering and being eaten by their predators. Conversely, a more convoluted (diffusive) motility pattern is advantageous for individuals with abundant prey, as this increases the chance of staying within a food‐rich patch and at the same time decreases the risk of encountering its own predators (Visser and Kiørboe 2006). Taylor (1991) developed an expression describing the time‐dependent transition from ballistic to diffusive motility for a continuous random walk:

(1) $$RMS=(2v^{2}\tau (t‐\tau {\left(1‐e^{‐t/\tau }))\right)}^{0.5}$$

where *v* is the swimming speed, *t* is time, and τ the decorrelation time. Hence, *l* = *v* x *τ* is the decorrelation length scale, a measure of directional persistence.

We further hypothesize that a larger fraction of the population will leave the surface to search for new surfaces when starved than when well fed. To get an index of the relative proportion of flagellates swimming in the water vs. on the surface, we computed the total number of observations of flagellates in the water column and on surfaces in 900 frames and considered the ratio between these two values as an index of the proportion of free‐swimming flagellates.

### 2.5 Flow Field Study

Neutrally buoyant latex beads (Thermo Scientific, Fremont CA, USA) at a final concentration of 0.01%–0.001% w/w (in water) were used as tracer particles to observe the flow surrounding the active predators. Beads of 0.3‐μm diameter were used for *Nib. kosolapovi*, and 0.5‐μm beads were used for the remaining species. To avoid aggregation, beads were first treated with bovine serum albumin (< 0.1 mg mL‐1) and sonicated until clump‐free. Tracer particles were gently added to a culture‐filled observation chamber, before sealing it with a coverslip. The behavior of the beads near surface‐associated specimens was video recorded and described.

### 2.6 Swimming Simulations

We did a conceptual simulation study of the effect of the vane‐and‐groove system on the swimming performance of the flagellate. We only considered the vane‐bearing posterior flagellum in these simulations. Simulations were performed using a generic excavate‐like model with a variable groove depth. The body dimensions are 5 × 2.5 × 2.5 μm. The planar beating pattern of the single flagellum is prescribed at a point of arclength s and time t by its tangent angle $\theta \left(s,t\right)$ relative to the body position

$$\theta \left(s,t\right)=R\sin\left(\frac{\mathrm{k\pi s}}{L}\right)\cos\left(2\mathrm{\pi ft}-\frac{\mathrm{\phi s}}{L}\right)$$

With $L=20\;\mu m,R=0.5\;\left(\mathrm{rad}\right),k=\frac{3\pi }{4}$ and ϕ=2π. We set the frequency f=1 so that one unit of time in the simulation corresponds to one beat cycle. The vane, of length 6.5μm and height 1μm, maintains a vertical position above the corresponding point on the flagellum. The depth d of the ellipse‐shaped groove floor is varied from d=0.0−2.5μm.

All simulations were performed using a recently developed Julia‐based framework for micro swimmer modeling and visualization (Cass and Wan in prep). Briefly, we approximate the force distribution using a finite set of regularized point forces or Stokeslets (Cortez 2001), and solved the Stokes' equation for the system by boundary integral methods (Pozrikidis 1992). All simulations use a regularization parameter of ϵ=0.1μm which approximates the flagellar radius. We employed a nearest‐neighbor discretization scheme (Smith 2018). For the body/flagellum we use N=1113/37 points for the force discretization and Q=5137/157 quadrature points.

## 3 Results

### 3.1 Morphology

**FIGURE 1:**
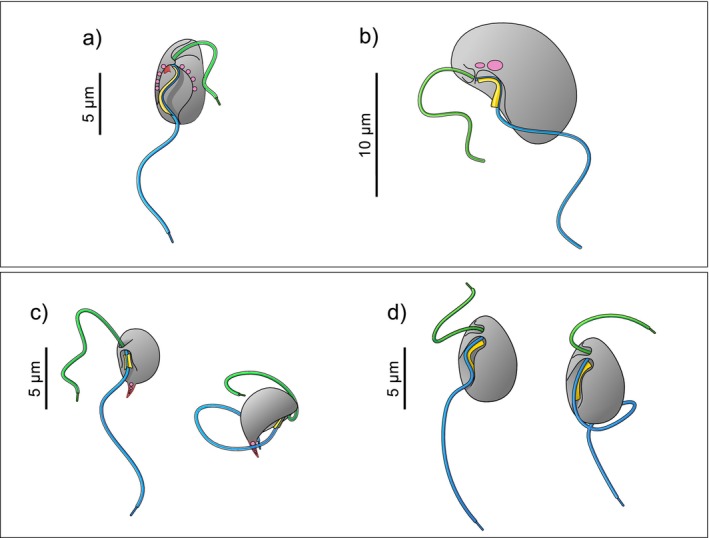
Schematic representation of the species of eukaryovorous flagellates: (a) *Neocolponema saponarium*, (b) *Colponema vietnamica*, (c) *Nibbleromonas kosolapovi*, and (d) *Nebulomonas marisrubri*. The posterior flagellum (blue) is equipped with one or two vanes (yellow) and beats in association with a more or less conspicuous ventral groove. The anterior flagellum (green) beats pointing posteriorly. These species have extrusive organelles (extrusomes) (pink) to permanently attach to their prey. For (c and d), the drawing on the left is the position of the flagella when prey searching, and on the right when they have captured prey. Note that we have not observed the vane; instead, references have been obtained from prior electron microscopy studies (Belyaev et al. 2024; Gigeroff et al. 2023; Mylnikov and Tikhonenkov 2009; Tikhonenkov et al. 2014, 2022). The orientation of the vane of *Colp. vietnamica* was not reported and therefore has been pictured the same as the other alveolate *Neo. saponarium*, while *Nib. kosolapovi* differs from the other species by having two very narrow vanes.

*Neocolponema saponarium* cells appear tear‐shaped, with a flattened lateral side and a tapered anterior end (Figure 1a). *Colponema vietnamica* (Figure 1b) cells are elongated and bean‐shaped, with a granulated surface and an evident straight lateral side in starved cells. *Nibbleromonas kosolapovi* cells are small, short, bean‐shaped, and with a thorn‐like structure protruding at the posterior end (Figure 1c). For all the above species, well‐fed cells become rounder. *Nebulomonas marisrubri* cells are oval to pyriform, with a tapered anterior end (Figure 1d). Well‐fed cells form a single large food vacuole at the posterior end, making this portion of the cell markedly wider than the anterior end.

*Neocolponema saponarium* has a conspicuous, broad ventral groove that opens at the posterior end. The remaining three species present less conspicuous and shorter ventral grooves. In particular, the groove of *Nib. kosolapovi* is barely visible under light microscopy and the groove of *Neb. marisrubri* is subtle and narrow (approximately 0.5 μm wide) (Video S4b). All four species have both flagella protruding from the anterior‐ventral portion of the cell (Figure 1). The posterior flagellum is approximately two body‐lengths long, points posteriorly, and beats closely to the ventral groove. The anterior flagellum, about a cell‐length long or more, also points backwards, beating in a three‐dimensional pattern along the straight lateral side of the cell.

**FIGURE 2:**
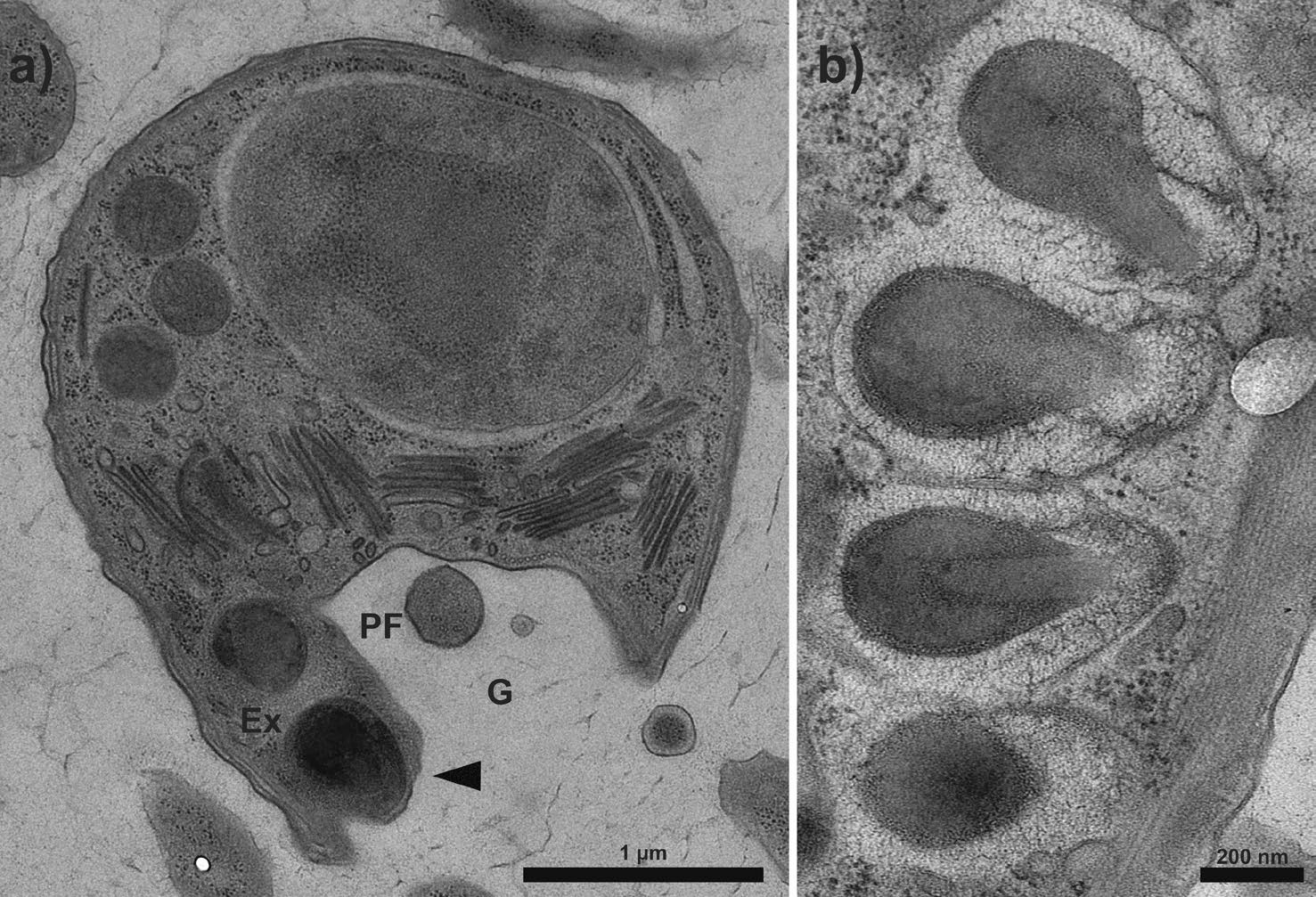
Transmission electron microscopy of two *Neocolponema saponarium* cells. (a) Whole cell transverse section through the extreme anterior end of the ventral groove (G), viewed from anterior end, showing position of an extrusome (Ex) within the tooth‐like structure (arrow). The posterior flagellum (PF) is sectioned very near to its insertion, before the start of the flagellar vane. (b) Four presumed reserve extrusomes showing shape and structure. Scale bars = 1 μm (a) and 200 nm (b).

Transmission electron microscopy of *Neo. saponarium* confirms the presence of several large flask‐shaped extrusomes with a central tube (Figure 2), similar to the extrusomes in *Colponema loxodes* (Mignot and Brugerolle 1975). One extrusome underlies a protrusion of the cell membrane at the anterior end of the ventral groove next to its right margin (Figure 2a), forming the ’tooth‐like structure’ previously identified by scanning electron microscopy (Gigeroff et al. 2023). A row of presumptive reserve extrusomes continues along the right wall of the groove (Figure 2b).

### 3.2 Swimming Behavior

**FIGURE 3:**
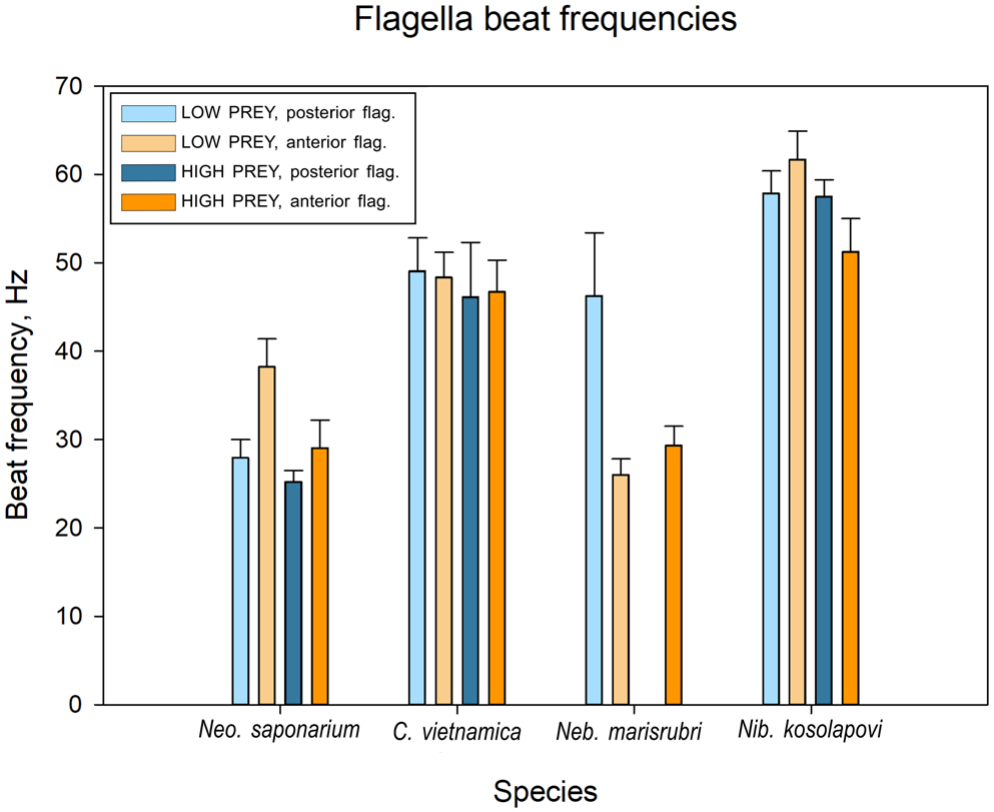
Beat frequencies (mean ± 95% CL) of *Neocolponema saponarium*, *Colponema vietnamica*, *Nibbleromonas kosolapovi* and *Nebulomonas marisrubri*, while moving on the surface. The external part of the posterior flagellum of *Neb. marisrubri* is attaching to the surface and not actively beating. Only statistically significant differences (non‐overlapping confidence limits) are emphasized in the text. [Data in Table S2].

*Neocolponema saponarium* and *C. vietnamica* have very similar swimming behaviors. The posterior flagellum beats in a plane parallel to the ventral groove floor, and the beat waves extend to the distal tip, forming one wavelength inside the groove and another ~3/4 wave with a larger amplitude outside. When swimming close to the surface, the ventral groove lays parallel to and faces the surface. Changes of direction are performed when the posterior flagellum slows down or pauses, anchoring the body while the anterior flagellum continues beating to reorientate the swimming trajectory (Videos S1a and S2a). The anterior flagellum of *Neo. saponarium* beats faster than the posterior flagellum, and both flagella beat faster in the absence than in the presence of prey (Figure 3). The flagella of *C. vietnamica* are almost synchronized and beat faster than in *Neo. saponarium* and independently of prey availability (Figure 3). For both species, the frequencies of the two flagella are correlated (Figure 3). The behavior of the flagella is similar between surface associated and freely swimming cells. When free‐swimming, both species rotate on their longitudinal axis (Videos S1b and S2b). While swimming near the surface, the cells do not rotate but have the ventral groove facing the surface with the posterior flagellum beating inside.

**FIGURE 4:**
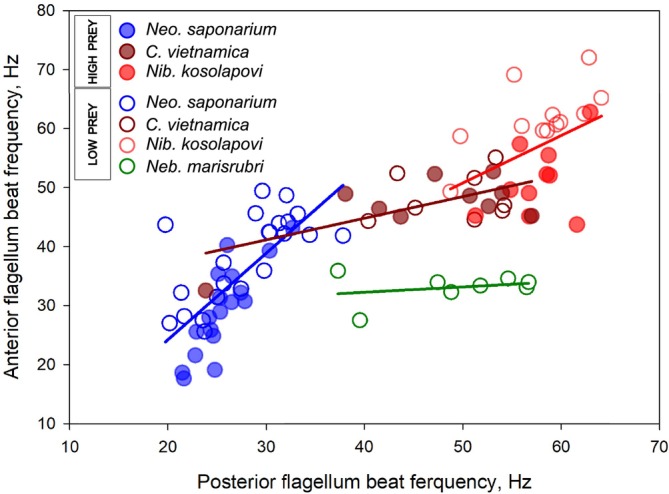
Relation between beat frequencies of posterior and anterior flagella in *Neocolponema saponarium*, *Colponema vietnamica*, *Nibbleromonas kosolapovi*, and *Nebulomonas marisrubri*. The correlation coefficients, respectively, are: *R* ^2^ = 0.53 (*p* < 0.0001), *R* ^2^ = 0.33 (*p* < 0.008), *R* ^2^ = 0.18 (*p* < 0.05), and *R* ^2^ = 0.07 (*p* > 0.05).

The posterior flagellum of *Nib. kosolapovi* beats freely, outside of the short groove, in a planar beat that is not perfectly aligned with the floor of the ventral groove, as the thorn‐like structure deviates the plane of the beat waves slightly sideways. Compared to the other species of this study, the beating pattern of the anterior flagellum of *Nib. kosolapovi* can resemble a power‐and‐recovery stroke, since the proximal end of the flagellum extends more anteriorly. The two flagella beat with similar high and correlated frequencies, except that the anterior flagellum has a slightly reduced beat frequency in the presence of food (Figures 3 and 4). The same flagellar kinematics serve as propulsion when moving near the surface (Video S3a) and in the bulk fluid (Video S3b). In both cases, the cell body rotates on its longitudinal axis; although the body rotation is not as smooth when swimming close to the surface, due to the interfering presence of the posterior ’thorn’. Occasionally, *Nib. kosolapovi* coils the posterior flagellum and swims erratically with the anterior flagellum. Alternatively, it can pause on the surface, with both flagella coiled. The functional significance of these behaviors remains unknown.

The distal end of the posterior flagellum of *Neb. marisrubri* is always contacting the surface when the cell is skidding along it. It only beats actively inside the ventral groove, exhibiting no beat waves once exiting the groove. The movement range of the anterior flagellum is broader than that of the other species, constantly beating while extending forward, sideways, or posteriorly. The anterior flagellum beats faster in the presence of food (Figure 3). Displacement on the surface is slow and with undefined trajectories, often loosely “anchoring” to a spot with the posterior flagellum and moving the cell in various directions (Video S4a). While anchored, the body irregularly rotates in both directions and is not permanently in contact with the surface. *Nebulomonas marisrubri* is highly associated with surfaces, and individuals were rarely observed in the bulk fluid.

### 3.3 Flow Through the Ventral Groove

We observed a flow through the groove of *Neo. saponarium*, *C. vietnamica* , and *Neb. marisrubri* cells that were swimming close to the surface (Videos S1c, S2c, and S4b). Tracer microspheres were drawn into the ventral groove once the particles were in very close proximity to the cell (< 1 μm). The beating anterior flagellum impeded many microspheres from entering the groove. For *Nib. kosolapovi*, tracer microparticles in the medium were not observed entering the ventral groove (Video S3c).

### 3.4 Motility Patterns of Well‐Fed Versus Starving Predators

**FIGURE 5:**
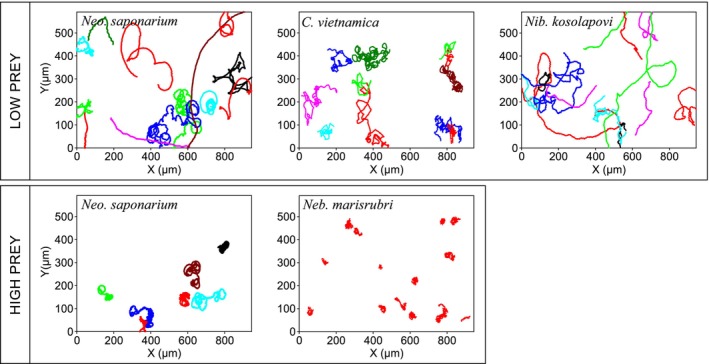
Motility patterns of four species of excavate‐like flagellates moving in association with a surface. In *Neocolponema saponarium*, motility patterns have been shown both in the presence and absence of prey. In the other species, motility patterns appear nearly independent of food availability and, therefore, they are only shown in one of the experimental food conditions (more data found in Table 1, Table S2, and Video S5). Note that different coloration was used only to distinguish overlapping tracks.

**FIGURE 6:**
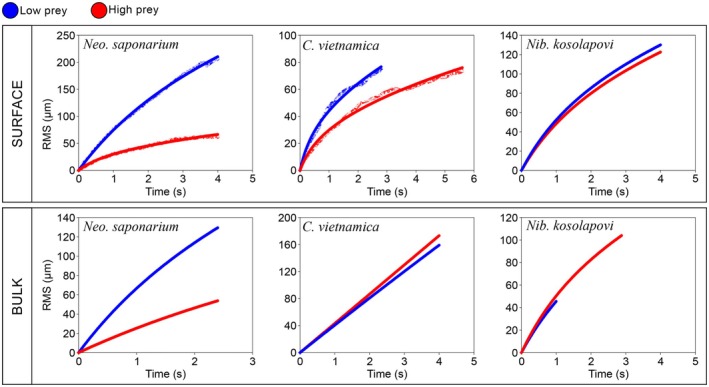
Taylor plots of motility patterns of *Neocolponema saponarium*, *Colponema vietnamica*, *Nibbleromonas kosolapovi*, and *Nebulomonas marisrubri*. Root‐mean square (RMS) distance traveled versus time of flagellates moving on a surface or in the bulk at high and low (no) prey availability. Parameters in Table 1. Fits of Equation (1) to the data are shown.

The individual swimming behaviors described above lead to different macroscale motility patterns, which may depend on the availability of food (Figure 5; Video S5). Thus, *Neo. saponarium* moves faster and with much more directional persistence in the absence than in the presence of food, both when swimming near a surface and in the bulk fluid. This is consistent with its higher flagellar beat frequencies in the absence of food (Figures 5, 6, Table S2). While many of the cells in the no‐food treatment have long, close‐to‐straight runs, most well‐fed cells have very convoluted tracks (Figure 5, Video S5a). When food is plentiful, most cells appear to be moving associated with the surface and in proximity to the prey, whilst the fraction of free‐swimming cells increases ten‐fold in the absence of food (Table 1).

**TABLE 1:** Summary of motility statistics for the four eukaryovorous species.

	High prey		Low prey	
	Surface	Bulk	Surface	Bulk
*Neocolponema saponarium*				
Speed (*v*, μm s^−1^)	25 ± 1	14 ± 0.2	44 ± 0.4	41 ± 0.4
Decorrelation length (*l*, μm)	12	43	82	62
Index fraction free swimming	0.034		0.31	
*Colponema vietnamica*				
Speed (*v*, μm s^−1^)	58 ± 4	43 ± 1	98 ± 7	41 ± 0.5
Decorrelation length (*l*, μm)	9	Infinite	11	Infinite
Index fraction free swimming	0.3		0.98	
*Nibbleromonas kosolapovi*				
Speed (*v*, μm s^−1^)	63 ± 10	65 ± 1	69 ± 0.4	60 ± 1
Decorrelation length (*l*, μm)	36	37	37	28
Index fraction free swimming	0.68		0.62	
*Nebulomonas marisrubri*				
Speed (*v*, μm s^−1^)	25 ± 12	—	37 ± 4	36 ± 5
Decorrelation length (*l*, μm)	—	—	—	—
Index fraction free swimming	~0		0.09	

*Note:* More detailed data in Table S1.

*Colponema vietnamica* is less surface associated and moves faster than *Neo. saponarium*, particularly in the absence of food, but its motility pattern and flagellar kinematics are otherwise nearly independent of food availability (Table 1, Figures 4, 5, 6, Video S5b). Surface associated cells have near‐diffusive motility patterns and a directional persistence similar to that of well‐fed *Neo. saponarium*, while freely swimming cells move near‐ballistically at the scale of the observations (decorrelation length ~ infinity), thus efficiently searching for new patches of food. *Nibbleromonas kosolapovi* moves at speeds similar to those of *C. vietnamica* , and its motility characteristics are independent of food availability and with intermediate directional persistence (Table 1; Figures 5 and 6, Video S5c). Finally, *Neb. marisrubri* is found almost entirely in association with a surface (Table 1). The cells may skid slowly over the surface but mainly stay near the same spot (Figure 5, Video S5d). Therefore, fitting Equation (1) to the data is not feasible.

### 3.5 Prey Encounter, Capture, and Handling

None of the four species generate a feeding current to capture food. Rather, they are “hunters” that collide with prey as they move around, without any clear evidence of remote detection.

**FIGURE 7:**
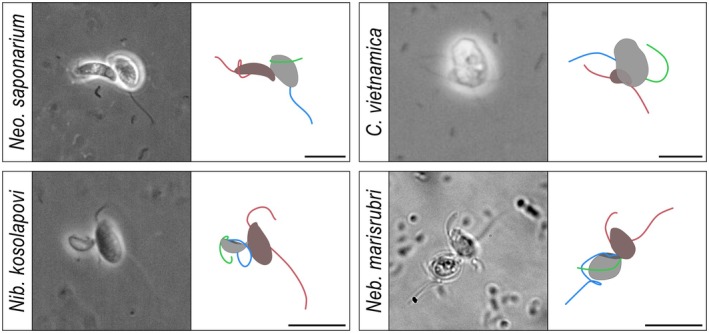
Body and flagella position of the four eukaryovorous predators (*Neocolponema saponarium*, *Colponema vietnamica, Nibbleromonas kosolapovi*, and *Nebulomonas marisrubri*) during prey capture. Prey is red‐brown, and predator is gray. Green and blue flagella are the anterior and posterior flagellum, respectively, of the predator. Note that when finally attached, the flagella of the predator are not involved in further handling of the prey. Scale bar 10 μm.

*Neocolponema saponarium* and *C. vietnamica* have similar prey encounter—capture and—handling behaviors (Figure 7; Videos S6 and S7). Random encounters elicit circling behavior, where the predator swims around the prey before possibly attaching and capturing it at the anterior end of the cell, where the prey is also phagocytized. During initial predator–prey attachment, the flagella of the predator are active and even increase beat frequency to push the predator against the prey. At some point, the flagella of the predator stop completely, plausibly indicating that the extrusomes located at the attachment site are discharged for final attachment to the prey. This final attachment is not rigid, as the prey is often observed changing body orientation without being released from the fixed attachment site. During this period, the flagella of the predator remain extended without engaging with the prey (Figure 7), and the prey gradually loses activity. *Colponema vietnamica* can restore the flagellar activity and start swimming during phagocytosis, whilst *Neo. saponarium* remains inactive until phagocytosis is complete. One case of cannibalism by *C. vietnamica* has been observed (Table S1).

To establish contact with a prey cell, *Nib. kosolapovi* increases the beating frequency of the posterior flagellum, pushing its posterior end of the cell towards the larger prey, and “landing” on it (Video S8). The predator then finally attaches to the prey with the posterior “thorn‐like” structure, likely aided by a discharged extrusome. At this point, the flagella stop in a coiled position (Figure 7). This attachment is flexible and allows the smaller predator to change its orientation relative to the prey. Eventually, the prey is immobilized, and *Nib. kosolapovi* leans over the prey to establish contact with its ventral groove. It then starts expanding over the prey, but only partially engulfs it through an “incomplete phagocytosis” process suggested to be like trogocytosis and termed “nibbling” by (Tikhonenkov et al. 2022).

*Nebulomonas marisrubri* attaches to prey at the anterior end of the cell with an inactive anterior flagellum, whilst beating the posterior flagellum inside the ventral groove (Video S9a). We often observed the predator releasing the prey after this initial attachment. The attachment becomes permanent once *Neb. marisrubri* performs a jolt and coils the posterior flagellum that then stops beating (Video S9b). The cell jolt is likely the predator firing an extrusome, as evidenced by one observation where an extrusome was fired at a distance (0.5 μm), and the prey was subsequently dragged towards the predator (Video S9c). Phagocytosis occurs from the anterior end of the ventral groove and prey of similar or smaller size are ingested entirely. However, when feeding on the large prey *Rhodomonas* sp., *Neb. marisrubri* only partially phagocytizes the cell. Until now, the predatory behavior of “biting off” or ‘nibbling’ a fraction of the prey has been reported for nibbleromonads (e.g., *Nib. kosolapovi*), but not nebulomonads (Tikhonenkov et al. 2022); thus its observation in *Neb. marisrubri* was unexpected.

### 3.6 Swimming Simulations

**FIGURE 8:**
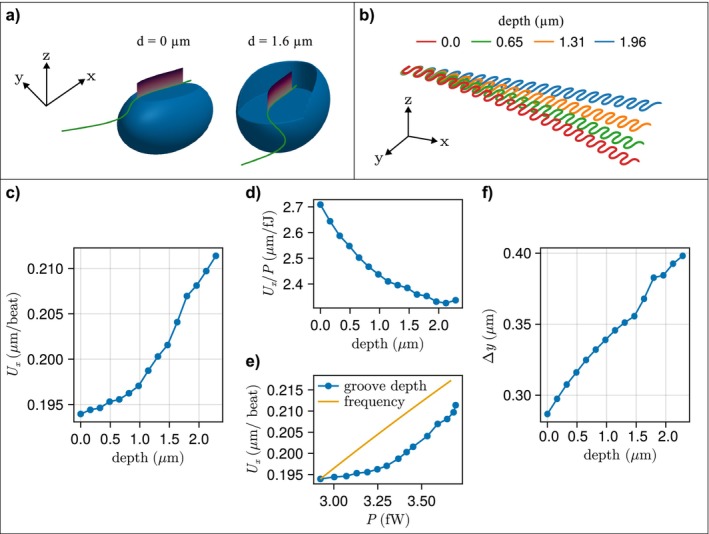
Swimming simulations. The model organism with either no groove (*d* = 0 μm) or a 1.6 μm deep groove (a) swims along an oscillating path that bends slightly in the Y‐direction, least so with the deep groove (b). The speed in the forward X‐direction (c) and the distance covered per femtojoule spent (d). Panel (e) compares the speed in the X‐direction of a vane‐and‐groove system with that of just increasing the beat frequency of a naked flagellum for increasing groove power consumption (and groove depth), and (f) shows 2 × amplitude of the sideways oscillation as a function of groove depth.

The presence of a groove increases the swimming speed by up to 7% (Figure 8c) but does so at the cost of a poorer fuel economy, by up to 16% (shorter distance per joule spent; Figure 8d), and it would be economically more advantageous to increase the beat frequency rather than having a groove (Figure 8e). The model organism swims forward (in X‐direction) along an oscillating path (oscillations in the Y‐direction) (Figure 8b,f), implying that the flagellate covers a gross distance that is up to 5 times longer than the net distance. While in the real organism this oscillation is compensated for by the activity of the anterior flagellum, it represents ’lost’ energy. This is the main reason for the poor fuel economy of the vane‐and‐groove system, and the reason that the fuel economy worsens with the depth of the groove as net to gross distance decreases by 19% between no groove and a deep groove.

## 4 Discussion

### 4.1 Prey Capture and Handling

The four phagotrophic flagellates examined here, two from Provora and two Alveolata, share major morphological features with “typical excavates”; namely a ventral groove (supported by a microtubular cytoskeleton of similar general architecture, at least in *Nibbleromonas kosolapovi*), and a vaned flagellum (with each vane supported by a paraxonemal lamellum) operating in association with the groove. However, the function of these similar features differ substantially: while most ‘typical excavates’ are attached to a surface to generate a feeding current from which small bacterial prey are harvested (Suzuki‐Tellier et al. 2024), the “excavate‐like” flagellates examined here do not generate a feeding current but are motile hunters that collide with prey. These different foraging strategies are adaptations to their different target prey. The ‘typical excavates’ forage on bacterial prey that are small enough to enter the ventral groove, that is, with a typical predator: prey volume ratio between 100 and 1000 (Figure S1). In contrast, the flagellates examined here are all eukaryvores, foraging on prey similar in size to the predator itself. Such large prey are not entrained in a feeding current and thus require a motile hunting strategy. Further, the excavate‐like species studied here, except *Nib. kosolapovi*, capture their prey with the anterior portion of the ventral groove, in contrast to the typical excavates that phagocytize their prey at the posterior end of the ventral groove (Tikhonenkov et al. 2014; Gigeroff et al. 2023; Suzuki‐Tellier et al. 2024). Also, while typical excavates continue to generate a feeding current while phagocytizing prey, the “excavate‐like” groups become non‐motile and have a long‐duration handling phase during which further prey cannot be captured, as found in some stramenopile flagellates and suspension feeding copepods (Figure S1).

In all four species examined here, collision with prey may lead to an initial attachment before attachment becomes permanent. Except for *Nib. kosolapovi* that attaches to its prey from the posterior end of the cell, initial attachments of the remaining three species occur at the anterior end of the cell while the posterior vaned flagellum of the predator is still actively beating inside the ventral groove (most clearly observed in *Nebulomonas marisrubri* in Video S9a). What is the mechanism of these initial attachments? The thrust generated by the beating posterior flagellum may push the predator towards the prey. Further, the two alveolates may orient their ventral groove towards the prey body during initial attachment. We speculate that, in this position the vaned flagellum beating in a groove may function like a “hydrodynamic glue”, much the same way that the parasitic flagellate *Giardia lamblia*, with a similar groove‐and‐vane arrangement, attaches to gut epithelia and other surfaces (Picou et al. 2024). At low Reynolds numbers, flow over a depression will follow the depression (creeping flow) and create a negative pressure or “suction force” that attaches *Giardia* to intestinal cells, or the eukaryovorous flagellate to its prey. While we have not been able to visualize the flow between the predator and the prey during initial attachment, the motion of particles demonstrates the fluid flow towards the bottom of the groove. It is the same creeping flow phenomenon that, in typical excavates, guides the flow over the ventral groove into the groove, where prey is captured.

The four eukaryovorous predators studied here are equipped with extrusive organelles—“extrusomes” (Figure 2; Tikhonenkov et al. 2014, 2022). These structures generally function in either defense or predation, in the latter case usually by being fired at the victim to paralyze it and/or adhere the prey to the predator (Hausmann 1978). Here, we suggest that extrusomes account for all final predator–prey attachments. We have direct evidence for only one species, *Neb. marisrubri*, where a fired extrusome dragged a prey cell towards the predator (Video S9c). However, in all species prey attachment occurs where these extrusive organelles are found, and a region of adhesive properties near the thorn‐like structure of *Nib. kosolapovi* has been described (Belyaev et al. 2024). The fact that prey remains mobile after being captured suggests that the primary role of the extrusive organelles is in attachment. A similar capture/attachment mechanism as that proposed here is found in dinoflagellates that similarly feed on large eukaryote prey (Hansen and Calado 1999).

Most bacterivorous flagellates handle prey with their flagella (Boenigk and Arndt 2000; Suzuki‐Tellier et al. 2022). However, the flagella of ‘typical excavates’ are not involved in this process. Instead, prey is usually handled by a “wave”: an outward bulge of the cell membrane that forms on the bottom of the groove and propagates posteriorly, moving captured prey with it to the posterior end of the groove, and there facilitating phagocytosis (Suzuki‐Tellier et al. 2023). No such ‘wave’ was found in the studied ‘excavate‐like’ species. Similar to typical excavates, we find that the flagella of the excavate‐like species examined here do not interact with the prey after final attachment, despite previous reports that the flagella in *Nibbleromonas piranha* (i.e., a congener of *Nib. kosolapovi*) are used to handle and immobilize the prey for ingestion (Belyaev et al. 2024). Rather, we observed that the flagella remain mostly inactive, and coiled (*Nib. kosolapovi* and *Neb. marisrubri*) or even pointing away from the prey (*Neocolponema saponarium* and *Colponema vietnamica*).

### 4.2 Motility Patterns

The species examined associated with surfaces almost entirely (*Neb. marisrubi*), mainly (*Nib. kosolapovi*) or for a substantial but varying fraction of the time (*Neo. saponarium* and *C. vietnamica* ) (Table 1). We cannot rule out that this is an artifact due to the prey in our preparations mainly being at the bottom of the observation chamber, but in nature, elevated prey concentrations are typically found near surfaces, for example, of marine snow (Simon et al. 2002) or in sediments. The two alveolates (*Neo. saponarium* and *C. vietnamica* ) were associated with the surface mainly when prey was abundant, but both moved into the water column when prey was scarce, which we interpret as a search for new prey patches. Similar behavioral adaptations to varying prey availability are found in other phagotrophic flagellates such as the stramenopiles *Pteridomonas danica*, *Spumellas* sp., and *Ochromonass* sp. (Christensen‐Dalsgaard and Fenchel 2004; Pfandl et al. 2004). Some of the species, most notably *Neo. saponarium*, showed additional adaptations to prey availability, by increasing flagellar beat frequencies, swimming speed and directional persistence in the absence of prey. These adaptations will increase the chance of encountering new (patches of) prey.

### 4.3 Clearance Rates

If we assume that the hunting excavate‐like flagellates encounter their prey randomly we can evaluate the volume of water searched for prey per unit time (~ maximum possible clearance rate). Because encounters are ballistic (decorrelation length scale > > prey size), the encounter volume rate is given by π(*R* + *r*)^2^ *v*, where *R* and *r* are, respectively, the radius of predator and prey, and *v* the swimming speed of the predator. Because to first order *R* ~ *r*, and *v* ~ 10*R* s^−1^ (Table 1; Table S1) this yields 40π*R* ^3^ s^−1^. If expressed relative to predator volume (4/3 π*R* ^3^), this yields a specific prey encounter volume rate of ca 2.5 × 10^6^ predator cell volumes per day. Clearance rates of this order of magnitude are required to maintain a population in the face of mortality in the plankton, and are similar to that found among planktonic predators, ranging from bacterivorous flagellates to fish (Kiørboe and Hirst 2014).

Ballistic encounter cannot be assumed for *Neb. marisrubri*, which moves on very convoluted paths and seems to search the same volume or area repeatedly (Figure 5). To survive, this species would likely need to live in an environment with a plentiful food supply, like marine snow or a sediment surface where prey density may be high.

### 4.4 Propulsion and the Role Vane‐and‐Groove Flagellar Arrangement

The two alveolate species swim in similar ways, with the posterior flagellum extending posteriorly beyond the ventral groove with a sperm‐like beat pattern (i.e., a near planar sinusoid wave with less than one full wave outside the groove), which is optimal for propulsion (Asadzadeh et al. 2023), and the anterior flagellum also pointing backwards and beating in a 3‐dimensional pattern. The combined action of the two flagella allows the cells to control their swimming direction. This propulsion mode is similar but different from that of typical excavates: the anterior flagellum of the latter also beats in a 3‐dimensional pattern, but with power‐and‐recovery strokes that typically begin ahead of the cell. Conversely, the posterior flagellum of *Nib. kosolapovi* beats close to the ventral groove but is poorly associated with it. The different flagellar behavior of *Nib. kosolapovi*, may be adapted to the presence of the posterior “thorn‐like” structure and to the distinctive way that it establishes the initial contact with their prey. It may also explain the continuous axial rotation observed, even when swimming along the surface.

The role of the vaned flagellum operating near a groove in the context of propulsion is unclear. For typical excavates that attach to the surface when foraging, we know that this arrangement reduces the sideways motion of fluid and rather directs it lengthwise in the groove to produce an efficient feeding current (Suzuki‐Tellier et al. 2024). However, according to our numerical experiment, the vane‐and‐groove system appears overall to be disadvantageous to propulsion because in the untethered organism it implies significant oscillation along the swimming path that must be compensated by the action of the anterior flagellum, and this problem increases with the depth of the groove. However, the cost of propulsion, ~3 fw (Figure 8), represents only a tiny fraction of the whole cell metabolism (~1%) (computed from data in Kiørboe and Hirst (2014)), and about 1% of the construction and operation cost of the entire cell in flagellates (Schavemaker and Lynch 2022). In addition, the differences between the different flagellar arrangements considered here are relatively small (< 20%), and particularly small for the excavate‐like cells that mostly have shallow grooves and narrow vanes. It is not obvious whether the extra costs of swimming due to the vane‐and‐groove arrangement are warranted by its role in attachment to prey (mainly in the two alveolates). Therefore, while the adaptive value—if any—of this morphology in the excavate‐like flagellates is unclear, the system is critical for efficient foraging in typical excavates.

### 4.5 Phylogenetic Implications

Despite belonging to different “supergroups” of eukaryotes, the excavate‐like species examined here have similar morphology and functional ecology: they swim and forage in similar ways. In contrast, even though sharing morphological features with the typical excavates, the functional ecology of the excavate‐like flagellates is distinctly different: they are hunters rather than feeding‐current feeders; they forage on large versus small prey; they are not attached when foraging; the prey is phagocytized in the anterior rather than the posterior end of the cell (groove); and prey contact and phagocytosis is facilitated by extrusomes rather than by a ‘wave’.

It is unclear from available morphological evidence to what extent the morphological similarities between typical excavates and these ‘excavate‐like’ eukaryvores represent homologies, which could thus represent ancient traits for the eukaryote tree of life; it is a possibility, however (Belyaev et al. 2024). The fact that similar morphologies function very differently in the typical excavates and these excavate‐like protists is consistent with a range of evolutionary scenarios. One possibility is that the vane and groove systems are directly homologous (and ancient) but have undergone an evolutionary change (or two such changes independently) in function from suspension‐feeding bacterivory to raptorial eukaryvory. At the other extreme, the vane and groove systems in alveolates and provorans could have evolved completely independently from that of typical excavates. Many intermediate hypotheses are plausible, whereby a groove involved in feeding is deeply homologous but the association with a vane‐bearing posterior flagellum represents convergence. Further studies of the detailed anatomy may throw light on this question. Irrespective, the evolution of deeply‐branching vane‐and‐groove‐bearing flagellates is of huge potential importance to understanding eukaryote evolution more generally; we assert that functional ecology should be taken into account alongside molecular phylogenetics, detailed ultrastructure, and studies of the compositions of cytoskeletal elements.

## Funding

This work was supported by Human Frontier Science Program (doi.org/10.52044/HFSP.RG0142024.pc.gr.194162). Simons Foundation (931976).

## Supporting information


**Video S1:** Swimming and flagellar behavior of *Neocolponema saponarium*. (a) Surface swimming. *Neocolponema saponarium* moving along the surface and redirecting the swimming path by changing the flagellar beating pattern. (b) Free swimming. *Neocolponema saponarium* free swimming with axial rotation of the body and changing direction. (c) Ventral groove flow. *Neocolponema saponarium* swimming along the surface and passing tracer particles (0.5 μm diameter) through the ventral groove.
**Video S2:** Swimming and flagellar behavior of *Colponema vietnamica*. (a) Surface swimming. *Colponema vietnamica* moving along the surface and redirecting the swimming path by changing the flagellar beating pattern. (b) Free swimming. *Colponema vietnamica* free swimming with axial rotation of the body. (c) Ventral groove flow. *Colponema vietnamica* swimming along the surface and passing tracer particles (0.5 μm diameter) through the ventral groove.
**Video S3:** Swimming and flagellar behavior of *Nibbleromonas kosolapovi*. (a) Surface swimming. *Nibbleromonas kosolapovi* moving along the surface with axial rotation of the body. Note that the swimming path is not smooth and interrupted by the thorn‐like structure at the posterior end of the cell. (b) Free swimming. *Nibbleromonas kosolapovi* free swimming with axial rotation of the body and changing direction. (c) Ventral groove flow. *Nibbleromonas kosolapovi* swimming along the surface in the presence of 0.3‐μm diameter tracer particles. The microparticles do not pass through the ventral groove, therefore there is no evident flow.
**Video S4:** Swimming and flagellar behavior of *Nebulomonas marisrubri*. (a) Surface skidding. *Nebulomonas marisrubri* moving along the surface. The end of the posterior flagellum remains in contact with the surface, while the body rotates. Skidding paths are not ballistic. (b) Ventral groove flow. Crossection view from the posterior end of the cell body of *Nebulomonas marisrubri*. A tracer particle (0.5 μm diameter) enters the ventral groove.
**Video S5:** Macroscale motility patterns. Well‐fed versus starving predators. (a) Motile behavior of *Neocolponema saponarium* cells swimming near a surface in the absence and presence of prey (*Novijibodo darinka*). The white dots are *Neo. saponarium* cells, and the smaller, darker dots are prey. Field of view is 945 × 591 mm^2^. At low prey density, the cells swim faster and perform longer loops (higher directional persistence). (b) Motile behavior of *Colponema vietnamica* cells swimming near a surface in the absence and presence of prey (*Parabodo caudatus*). The big cells are the predator, and the small, non‐conspicuous cells are prey. (c) Motile behavior of *Nibbleromonas kosolapovi* cells swimming near a surface in the absence and presence of prey (*Rhodomonas* sp.). The small, motile cells are the predator, while the bigger, non‐motile cells are prey. (d) Motile behavior of *Nebulomonas marisrubri* cells swimming near a surface in the absence and presence of prey (*Rhodomonas* sp.). The motile, bright cells are the predator, and the non‐motile, bigger, bright cells are prey (only present on the right panel). Note that, on the left panel, there are two differentiated cell sizes of *Neb. marisrubi*.
**Video S6:** Prey capture. The posterior flagellum of *Neocolponema saponarium* increases its beat frequency when entering in contact with the prey. After various bumping encounters, the predator attaches to the prey (*Novijibodo darinka*). Upon attachment, the prey gradually loses activity.
**Video S7:** Prey capture. *Colponema vietnamica* bumps into its prey (*Parabodo caudatus*) several times before “finally” attaching to it. The flagella of the predator do not engage in prey handling. The prey gradually loses activity after attempting to be released.
**Video S8:** Prey capture. *Nibbleromonas kosolapovi* “lands” on the prey (*Procryptobia sorokini*) by increasing the beat frequencies and slightly modifying the flagellar position, while contacting the prey with the thorn‐like structure. Once the predator attaches to the prey with the thorn‐like structure, it coils and immobilizes the flagella. In this case, the prey remains active and *Nib. kosolapovi* “rides” on its next meal.
**Video S9:** Prey capture by *Nebulomonas marisrubri*. (a) Initial attachment. *Nebulomonas marisrubri* attaches to a *Rhodomonas* sp. cell by the anterior end of the cell (initial attachment). The anterior flagellum stops beating while the posterior flagellum beats inside the ventral groove. This attachment is not permament, as the predator releases the prey and swims away after a while. (b) Prey capture. *Nebulomonas marisrubri* captures its prey (*Procryptobia sorokini*). The predator establishes contact with the prey, and its anterior flagellum stops beating, whilst its posterior flagellum remains active inside the ventral groove (initial attachment). Then, the posterior flagellum rapidly coils, supposedly, when extrusomes are fired towards the prey. *Nebulomonas marisrubri* remains in the coiled position, permanently attached to a still active prey. (c) Prey capture. *Nebulomonas marisrubri* captures its prey (*Procryptobia sorokini*). The jolt and coiling of the posterior flagellum of *Neb. marisrubri* happens at a distance from the still active prey. Then, the prey is gradually “dragged” towards the predator until the two cell bodies establish contact. This suggests that extrusomes can be effective, despite not being in contact, and confirms the adhesive function of these extrusive structures.
**Table S1:** Predator and prey cell dimensions. Averaged cell measurements (with standard deviations) and the predator–prey volume ratios (assuming ellipsoidal cell bodies) of *Neocolponema saponarium*, *Colponema vietnamica*, *Nibbleromonas kosolapovi*, and *Nebulomonas marisrubri* and their ingested prey. *N* = number of observations. Note that cannibalism has been observed once by *C. vietnamica* .
**Table S2:** Beat frequencies—mean ± 95% CL (number of observations in parentheses). The external part of the posterior flagellum of *Nebulomonas marisrubri* is attached to the surface and not actively beating.
**Table S3:** *Neocolponema saponarium*. Number of swimming tracks analyzed and total number of observations of flagellates (“spots”) in the four treatments as well as the index of fraction of free‐swimming flagellates with and without food.
**Table S4:** *Nibbleromonas kosolapovi*. Number of swimming tracks analyzed and total number of observations of flagellates (“spots”) in the four treatments as well as the index of fraction of free‐swimming flagellates with and without food.
**Table S5:** *Colponema vietnamica*. Number of swimming tracks analyzed and total number of observations of flagellates (“spots”) in the four treatments as well as the index of fraction of free‐swimming flagellates with and without food.
**Table S6:** *Nebulomonas marisrubri*. Number of swimming tracks analyzed and total number of observations of flagellates (“spots”) in the four treatments as well as the index of fraction of free‐swimming flagellates with and without food.
**Figure S1:** Handling times in flagellates and copepods as a function of relative prey size. Data on bacteriovorous flagellates (Stramenopila) is from Suzuki‐Tellier et al. (2022) and data on copepods is from Ryderheim et al. (2023).

## Data Availability

The data that support the findings of this study are available from the corresponding author upon reasonable request.

## References

[jeu70084-bib-0001] Asadzadeh, S. S. , J. H. Walther , and T. Kiørboe . 2023. “Conflicting Roles of Flagella in Planktonic Protists: Propulsion, Resource Acquisition, and Stealth.” PRX Life 1, no. 1: 13002. 10.1103/PRXLife.1.013002.

[jeu70084-bib-0002] Belyaev, A. O. , S. A. Karpov , P. J. Keeling , and D. V. Tikhonenkov . 2024. “The Nature of ‘Jaws’: A New Predatory Representative of Provora and the Ultrastructure of Nibbling Protists.” Open Biology 14, no. 12: 240158. 10.1098/rsob.240158. 39689855 PMC11651884

[jeu70084-bib-0003] Boenigk, J. , and H. Arndt . 2000. “Particle Handling During Interception Feeding by Four Species of Heterotrophic Nanoflagellates.” Journal of Eukaryotic Microbiology 47, no. 4: 350–358. 10.1111/j.1550-7408.2000.tb00060.x. 11140448

[jeu70084-bib-0004] Brugerolle, G. 2006. “Description of a New Freshwater Heterotrophic Flagellate Sulcomonas Lacustris Affiliated to the Collodictyonids.” Acta Protozoologica 45, no. 2: 175–182.

[jeu70084-bib-0005] Brugerolle, G. u. y. , G. Bricheux , H. Philippe , and G. Coffe . 2002. “Collodictyon Triciliatum and *Diphylleia rotans (= Aulacomonas submarina)* Form a New Family of Flagellates (Collodictyonidae) With Tubular Mitochondrial Cristae That Is Phylogenetically Distant From Other Flagellate Groups.” Protist 153, no. 1: 59–70. 10.1078/1434-4610-00083. 12022276

[jeu70084-bib-0006] Cass, J. F. , and K. Y. Wan . in prep. Open‐Source Simulation Environment for Cilia‐Driven Motility.

[jeu70084-bib-0007] Christensen‐Dalsgaard, K. K. , and T. o. m. Fenchel . 2004. “Complex Flagellar Motions and Swimming Patterns of the Flagellates Paraphysomonas Vestita and Pteridomonas Danica.” Protist 155, no. 1: 79–87. 10.1078/1434461000166. 15144060

[jeu70084-bib-0008] Cortez, R. 2001. “The Method of Regularized Stokeslets.” SIAM Journal on Scientific Computing 23, no. 4: 1204–1225. 10.1137/S106482750038146X.

[jeu70084-bib-0009] Derelle, R. , G. Torruella , V. Klimeš , et al. 2015. “Bacterial Proteins Pinpoint a Single Eukaryotic Root.” Proceedings of the National Academy of Sciences 112, no. 7: E693–E699. 10.1073/pnas.1420657112. PMC4343179 25646484

[jeu70084-bib-0010] Gigeroff, A. S. , Y. Eglit , and A. G. B. Simpson . 2023. “Characterisation and Cultivation of New Lineages of Colponemids, a Critical Assemblage for Inferring Alveolate Evolution.” Protist 174, no. 2: 125949. 10.1016/j.protis.2023.125949. 37019068

[jeu70084-bib-0011] Hansen, P. J. , and A. J. Calado . 1999. “Phagotrophic Mechanisms and Prey Selection in Free‐Living Dinoflagellates.” Journal of Eukaryotic Microbiology 46, no. 4: 382–389. 10.1111/j.1550-7408.1999.tb04617.x.

[jeu70084-bib-0012] Hausmann, K. 1978. “Extrusive Organelles in Protists.” In International Review of Cytology, vol. 52, 197–276. Academic Press. 10.1016/S0074-7696(08)60757-3. 418022

[jeu70084-bib-0013] He, D. , O. Fiz‐Palacios , C.‐J. Fu , J. Fehling , C.‐C. Tsai , and S. L. Baldauf . 2014. “An Alternative Root for the Eukaryote Tree of Life.” Current Biology 24, no. 4: 465–470. 10.1016/j.cub.2014.01.036. 24508168

[jeu70084-bib-0014] Heiss, A. A. , G. Walker , and A. G. B. Simpson . 2011. “The Ultrastructure of *Ancyromonas*, a Eukaryote Without Supergroup Affinities.” Protist 162, no. 3: 373–393. 10.1016/j.protis.2010.08.004. 21420357

[jeu70084-bib-0015] Heiss, A. A. , G. Walker , and A. G. B. Simpson . 2013. “The Microtubular Cytoskeleton of the Apusomonad Thecamonas, a Sister Lineage to the Opisthokonts.” Protist 164, no. 5: 598–621. 10.1016/j.protis.2013.05.005. 23872341

[jeu70084-bib-0016] Janouškovec, J. , D. V. Tikhonenkov , K. V. Mikhailov , et al. 2013. “Colponemids Represent Multiple Ancient Alveolate Lineages.” Current Biology 23, no. 24: 2546–2552. 10.1016/j.cub.2013.10.062. 24316202

[jeu70084-bib-0017] Kiørboe, T. , and A. G. Hirst . 2014. “Shifts in Mass Scaling of Respiration, Feeding, and Growth Rates Across Life‐Form Transitions in Marine Pelagic Organisms.” American Naturalist 183, no. 4: E118–E130. 10.1086/675241. 24642502

[jeu70084-bib-0018] Mesbah, N. M. , D. B. Hedrick , A. D. Peacock , M. Rohde , and J. Wiegel . 2007. “ *Natranaerobius thermophilus* Gen. Nov., sp. Nov., a Halophilic, Alkalithermophilic Bacterium From Soda Lakes of the Wadi an Natrun, Egypt, and Proposal of Natranaerobiaceae Fam. Nov. and Natranaerobiales Ord. Nov.” International Journal of Systematic and Evolutionary Microbiology 57, no. 11: 2507–2512. 10.1099/ijs.0.65068-0. 17978210

[jeu70084-bib-0019] Mignot, J. P. , and G. Brugerolle . 1975. “Étude ultrastructurale du flagelle phagotrophie Colponema loxodes.” Protist 11, no. 4: 429–444.

[jeu70084-bib-0020] Mylnikov, A. , and D. Tikhonenkov . 2009. “The New Alveolate Carnivorous Flagellate Colponema Marisrubri sp. N. (Colponemida, Alveolata) From the Red Sea.” Zoologicheskiĭ Zhurnal 88: 1163–1169.

[jeu70084-bib-0021] Packer, J. A. , D. Zavadska , E. J. Weston , Y. Eglit , D. J. Richter , and A. G. B. Simpson . 2025. “Characterization of Allobodo Yubaba sp. Nov. and Novijibodo Darinka Gen. Et sp. Nov., Cultivable Free‐Living Species of the Phylogenetically Enigmatic Kinetoplastid Taxon Allobodonidae.” Journal of Eukaryotic Microbiology 72, no. 1: e13072. 10.1111/jeu.13072. 39868642 PMC11771631

[jeu70084-bib-0022] Pfandl, K. , T. Posch , and J. Boenigk . 2004. “Unexpected Effects of Prey Dimensions and Morphologies on the Size Selective Feeding by Two Bacterivorous Flagellates (Ochromonas sp. and Spumella sp.).” Journal of Eukaryotic Microbiology 51, no. 6: 626–633. 10.1111/j.1550-7408.2004.tb00596.x. 15666719

[jeu70084-bib-0023] Picou, T. J. , H. Luo , R. J. Polackwich , et al. 2024. “A Novel Mechanism of Microbial Attachment: The Flagellar Pump of *Giardia lamblia* .” PNAS Nexus 3, no. 12: pgae545. 10.1093/pnasnexus/pgae545. 39660061 PMC11631216

[jeu70084-bib-0024] Pozrikidis, C. 1992. Boundary Integral and Singularity Methods for Linearized Viscous Flow. Cambridge University Press. 10.1017/CBO9780511624124.

[jeu70084-bib-0025] Ryderheim, F. , U. H. Thygesen , and T. Kiørboe . 2023. “Short Handling Times Allow for Active Prey Selection in Suspension Feeding Copepods.” Limnology and Oceanography 68, no. 4: 891–901. 10.1002/lno.12317.

[jeu70084-bib-0026] Schavemaker, P. E. , and M. Lynch . 2022. “Flagellar Energy Costs Across the Tree of Life.” eLife 11: e77266. 10.7554/eLife.77266. 35881430 PMC9323006

[jeu70084-bib-0027] Schneider, C. A. , W. S. Rasband , and K. W. Eliceiri . 2012. “NIH Image to ImageJ: 25 Years of Image Analysis.” Nature Methods 9, no. 7: 7. 10.1038/nmeth.2089. PMC5554542 22930834

[jeu70084-bib-0028] Serif (Europe) Ltd . 1987. Affinity Photo (Version 2.6.5)[Computer Software]. https://store.serif.com/update/windows/photo/1/.

[jeu70084-bib-0029] Simon, M. , H.‐P. Grossart , B. Schweitzer , and H. Ploug . 2002. “Microbial Ecology of Organic Aggregates in Aquatic Ecosystems.” Aquatic Microbial Ecology 28: 175–211. 10.3354/ame028175.

[jeu70084-bib-0030] Simpson, A. G. B. 2003. “Cytoskeletal Organization, Phylogenetic Affinities and Systematics in the Contentious Taxon Excavata (Eukaryota).” International Journal of Systematic and Evolutionary Microbiology 53, no. 6: 1759–1777. 10.1099/ijs.0.02578-0. 14657103

[jeu70084-bib-0031] Smith, D. J. 2018. “A Nearest‐Neighbour Discretisation of the Regularized Stokeslet Boundary Integral Equation.” Journal of Computational Physics 358: 88–102. 10.1016/j.jcp.2017.12.008.

[jeu70084-bib-0032] Suzuki‐Tellier, S. , A. Andersen , and T. Kiørboe . 2022. “Mechanisms and Fluid Dynamics of Foraging in Heterotrophic Nanoflagellates.” Limnology and Oceanography 67, no. 6: 1287–1298. 10.1002/lno.12077.

[jeu70084-bib-0033] Suzuki‐Tellier, S. , T. Kiørboe , and A. G. B. Simpson . 2023. “The Function of the Feeding Groove of ‘Typical Excavate’ Flagellates.” Journal of Eukaryotic Microbiology 71: e13016. 10.1111/jeu.13016. 38108228

[jeu70084-bib-0034] Suzuki‐Tellier, S. , F. Miano , S. S. Asadzadeh , A. G. B. Simpson , and T. Kiørboe . 2024. “Foraging Mechanisms in Excavate Flagellates Shed Light on the Functional Ecology of Early Eukaryotes.” Proceedings of the National Academy of Sciences 121, no. 22: e2317264121. 10.1073/pnas.2317264121. PMC11145212 38781211

[jeu70084-bib-0041] Taylor, G. I. 1991. “Diffusion by Continous Movement.” Proceedings of the London Mathematical Society 20: 196–212.

[jeu70084-bib-0035] Tikhonenkov, D. V. 2020. “Predatory Flagellates—The New Recently Discovered Deep Branches of the Eukaryotic Tree and Their Evolutionary and Ecological Significance.” Protistology 14, no. 1: 15–22. 10.21685/1680-0826-2020-14-1-2.

[jeu70084-bib-0036] Tikhonenkov, D. V. , J. Janouškovec , A. P. Mylnikov , et al. 2014. “Description of Colponema Vietnamica sp.n. and Acavomonas Peruviana n. Gen. N. Sp., Two New Alveolate Phyla (Colponemidia Nom. Nov. and Acavomonidia Nom. Nov.) and Their Contributions to Reconstructing the Ancestral State of Alveolates and Eukaryotes.” PLoS One 9, no. 4: e95467. 10.1371/journal.pone.0095467. 24740116 PMC3989336

[jeu70084-bib-0037] Tikhonenkov, D. V. , K. V. Mikhailov , R. M. R. Gawryluk , et al. 2022. “Microbial Predators Form a New Supergroup of Eukaryotes.” Nature 612, no. 7941: 7941. 10.1038/s41586-022-05511-5. 36477531

[jeu70084-bib-0042] Visser, A. W. , and T. Kiørboe . 2006. “Plankton Motility Patterns and Encounter Rates.” Oecologia 148: 538–546. 10.1007/s00442-006-0385-4. 16586112

[jeu70084-bib-0038] Williamson, K. , L. Eme , H. Baños , et al. 2025. “A Robustly Rooted Tree of Eukaryotes Reveals Their Excavate Ancestry.” Nature 640, no. 8060: 974–981. 10.1038/s41586-025-08709-5. 40074902

[jeu70084-bib-0039] Yubuki, N. , and B. S. Leander . 2013. “Evolution of Microtubule Organizing Centers Across the Tree of Eukaryotes.” Plant Journal: For Cell and Molecular Biology 75, no. 2: 230–244. 10.1111/tpj.12145. 23398214

[jeu70084-bib-0040] Yurchenko, V. , J. Votýpka , M. Tesarová , et al. 2014. “Ultrastructure and Molecular Phylogeny of Four New Species of Monoxenous Trypanosomatids From Flies (Diptera: Brachycera) With Redefinition of the Genus Wallaceina.” Folia Parasitologica 61, no. 2: 97–112. 24822316

