# Supplementary material for: Eukaryovorous Predation in Evolutionarily Significant Excavate‐Like Flagellates
Source: J Eukaryot Microbiol. 2026 Apr 29;73:e70084. doi: 10.1111/jeu.70084 (PMC13128348; doi:10.1111/jeu.70084)
Supplement: Supplementary file 1 — Video S1: Swimming and flagellar behavior of Neocolponema saponarium. (a) Surface swimming. Neocolponema saponarium moving along the surface and redirecting the swimming path by changing the flagellar beating pattern. (b) Free swimming. Neocolponema saponarium free swimming with axial rotation of the body and changing direction. (c) Ventral groove flow. Neocolponema saponarium swimming along the surface and passing tracer particles (0.5 μm diameter) through the ventral groove. Video S2: Swimming and flagellar behavior of Colponema vietnamica. (a) Surface swimming. Colponema vietnamica moving along the surface and redirecting the swimming path by changing the flagellar beating pattern. (b) Free swimming. Colponema vietnamica free swimming with axial rotation of the body. (c) Ventral groove flow. Colponema vietnamica swimming along the surface and passing tracer particles (0.5 μm diameter) through the ventral groove. Video S3: Swimming and flagellar behavior of Nibbleromonas kosolapovi. (a) Surface swimming. Nibbleromonas kosolapovi moving along the surface with axial rotation of the body. Note that the swimming path is not smooth and interrupted by the thorn‐like structure at the posterior end of the cell. (b) Free swimming. Nibbleromonas kosolapovi free swimming with axial rotation of the body and changing direction. (c) Ventral groove flow. Nibbleromonas kosolapovi swimming along the surface in the presence of 0.3‐μm diameter tracer particles. The microparticles do not pass through the ventral groove, therefore there is no evident flow. Video S4: Swimming and flagellar behavior of Nebulomonas marisrubri. (a) Surface skidding. Nebulomonas marisrubri moving along the surface. The end of the posterior flagellum remains in contact with the surface, while the body rotates. Skidding paths are not ballistic. (b) Ventral groove flow. Crossection view from the posterior end of the cell body of Nebulomonas marisrubri. A tracer particle (0.5 μm diameter) enters the ventral gr [file JEU-73-e70084-s001.zip › jeu70084-sup-0008-supinfo.docx]

**SUPPORTING INFORMATION**

|  | | | Predator | | Prey | | Predator : Prey |
| --- | --- | --- | --- | --- | --- | --- | --- |
|  |  |  | Major axis | Minor axis | Major axis | Minor axis |  |
| Predator species | Prey species | N | µm | µm | µm | µm | µm^3^/µm^3^ |
| *Neo. saponarium* | *Novijibodo darinka* | 10 | 7.8 ± 0.7 | 4.8 ± 0.7 | 9.6 ± 1.7 | 2.8 ± 0.6 | 2.4 |
| *C. vietnamica* | *C. vietnamica* | 1 | 11.1 | 9.0 | 10.7 | 8.5 | 1. 2 |
| *C. vietnamica* | *Parabodo caudatus* | 5 | 13.4 ± 2.1 | 7.9 ± 1.9 | 6.4 ± 0.5 | 3.9 ± 0.3 | 8.54 |
| *C. vietnamica* | *Spumella* sp. | 4 | 10.9 ± 1.2 | 8.3 ± 0.5 | 7.9 ± 0.2 | 5.4 ± 1.2 | 3.3 |
| *Nib. kosolapovi* | *Rhodomonas* sp. | 1 | 3.6 | 2.4 | 12.2 | 7.9 | 0.03 |
| *Nib. kosolapovi* | *Procrybtobia* *sorokini* | 3 | 4.3 ± 0.5 | 2.2 ± 0.1 | 7.3 ± 0.6 | 2.9 ±0.2 | 0.3 |
| *Neb. marisrubri* | *Procrybtobia* *sorokini* | 3 | 6.6 ± 1.0 | 3.9 ± 0.6 | 6.4 ± 0.6 | 3.2 ± 0.5 | 1.6 |
| *Neb. marisrubri* | Unidentified flagellate | 1 | 6.2 | 3.7 | 2.7 | 1.8 | 9.1 |
| *Neb. marisrubri* | *Rhodomonas* sp. | 1 | 7.3 | 3.9 | 8.7 | 7.1 | 0.3 |

**Table S1**. **Predator and prey cell dimensions.** Averaged cell measurements (with standard deviations) and the predator-prey volume ratios (assuming ellipsoidal cell bodies) of *Neocolponema saponarium*, *Colponema vietnamica*, *Nibbleromonas kosolapovi,* and *Nebulomonas marisrubri* and their ingested prey. N = number of observations. Note that cannibalism has been observed once by *C. vietnamica*.

|  | No food | | With food | |
| --- | --- | --- | --- | --- |
|  | Posterior | Anterior | Posterior | Anterior |
| *Neo. saponarium* | 28.0 + 2.0 (24) | 38.3 + 3.1 (23) | 25.3 + 1.2 (21) | 29.1 + 3.1 (19) |
| *C. vietnamica* | 49.1 + 3.7 (8) | 48.4 + 2.8 (8) | 46.2+6.1 (10) | 46.8+3.5 (10) |
| *Neb. marisrubri* | 46.3 + 7.1 (9) | 26.1 + 1.7 (30) | No data | 29.4 + 2.1 (13) |
| *Nib. kosolapovi* | 57.9 + 2.5 (13) | 61.7 + 3.2 (12) | 57.5 + 1.9 (11) | 51.3 + 3.7 (10) |

**Table S2.** Beat frequencies – mean + 95 % CL (number of observations in parentheses). The external part of the posterior flagellum of *Nebulomonas marisrubri* is attached to the surface and not actively beating.

|  | Surface | | Free water | |
| --- | --- | --- | --- | --- |
|  | No food | With food | No food | With food |
| # tracks | 130 | 108 | 133 | 113 |
| Total # frames | 5400 | 5400 | 5400 | 5400 |
| Total number of observations (spots) | 38719/5400=  7.17 | 66711/5400=  12.35 | 12104/5400=  2.24 | 2282/5400=  0.42 |
| Index fraction free swimming | No food: 0.31 | | With food: 0.034 | |

**Table S3.** *Neocolponema saponarium*. Number of swimming tracks analyzed and total number of observations of flagellates (‘spots’) in the four treatments as well as the index of fraction of free-swimming flagellates with and without food.

|  | Surface | | Free water | |
| --- | --- | --- | --- | --- |
|  | No food | With food | No food | With food |
| # tracks | 182 | 224 | 687 | 138 |
| # of frames | 5400 | 18000 | 3600 | 4500 |
| Total number of observations/frame (spots) | 64288/5400=  11.9 | 96969/18000=  5.38 | 29683/3600=  7.41 | 16653/4500=  3.70 |
| Index fraction free swimming | No food:0.62 | | With food: 0.68 | |

**Table S4.** *Nibbleromonas kosolapovi.* Number of swimming tracks analyzed and total number of observations of flagellates (‘spots’) in the four treatments as well as the index of fraction of free-swimming flagellates with and without food.

|  | Surface | | Free water | |
| --- | --- | --- | --- | --- |
|  | No food | With food | No food | With food |
| # tracks | 14 | 55 | 95 | 225 |
| # of frames | 5400 | 5400 | 5400 | 5400 |
| Total number of observations/frame (spots) | 5082/5400=  0.94 | 26416/5400=  4.89 | 4944/5400=  0.92 | 7880/5400=  1.45 |
| Index fraction free swimming (corrected for # of frames) | No food: 0.98 | | With food: 0.30 | |

**Table S5.** *Colponema vietnamica*. Number of swimming tracks analyzed and total number of observations of flagellates (‘spots’) in the four treatments as well as the index of fraction of free-swimming flagellates with and without food.

|  | Surface | | Free water | |
| --- | --- | --- | --- | --- |
|  | No food | With food | No food | With food |
| # tracks | 465 | 262 |  | ~0 |
| # of frames | 5400 | 5400 | 5400 | 5400 |
| Total number of observations/frame (spots) | 285610  /5400=  52.9 | 149607  /5400=  27.7 | 25973  /5400=  4.80 | 0/5400=  0 |
| Index fraction free swimming | No food: 0.09 | | With food: ~0 | |

**Table S6.** *Nebulomonas marisrubri*. Number of swimming tracks analyzed and total number of observations of flagellates (‘spots’) in the four treatments as well as the index of fraction of free-swimming flagellates with and without food.

**Figure S1**. Handling times in flagellates and copepods as a function of relative prey size. Data on bacteriovorous flagellates (Stramenopila) is from Suzuki-Tellier et al. (2022) and data on copepods is from Ryderheim et al. (2023).
